# Viral Culture in Hospitalized Congregate Care Patients With Prolonged SARS-CoV-2 Viral RNA Detection

**DOI:** 10.1093/geroni/igab046.2718

**Published:** 2021-12-17

**Authors:** Thilaka Arunachalam, Amit Singh, Kathleen Stellrecht, Sarah Elmendorf, Tarani K Barman, Michael Robek, Dennis W Metzger, Michael Waxman

**Affiliations:** 1 Albany Medical College, Albany, New York, United States; 2 Albany Medical Center, ALBANY, New York, United States; 3 Albany Medical Center, Albany, New York, United States; 4 Albany Medical Center, Albany Medical Center, New York, United States; 5 Albany Medical Disease, Albany, New York, United States; 6 Albany Medical College, Albany Medical College, New York, United States

## Abstract

Prolonged detection of SARS-CoV-2 viral RNA has been observed in hospitalized congregate care patients following resolution of clinical symptoms. It is unknown whether patients with persistent PCR positivity pose a risk for COVID-19 transmission. The purpose of this study was to examine the results of serial PCR testing, viral load, and viral culture in patients awaiting discharge prior to a negative PCR test. We sampled 14 patients who were admitted from skilled nursing and/or rehabilitation facilities to a large academic medical center, had clinical signs and symptoms of COVID-19, and had multiple PCR-positive tests separated by at least 14 days. PCR-positive nasopharyngeal swabs were obtained from each patient for viral load quantification and viral culture. The mean age of patients was 72.5 years (55 – 92), with a mean peak SOFA score of 5.6 (1 – 11). Patients were hospitalized for a mean of 37.0 days (25 – 60). RNA was detected by PCR for a mean of 32.9 days (19 – 47). Mean viral load for the first PCR-positive nasopharyngeal swab collected at our hospital was 5.81 genomic copies/mL (2.12 – 9.72). Viral load decreased significantly with days from clinical symptom onset (R = -0.69, 95% CI, -0.80 – -0.55). Four out of 28 samples grew active virus via culture, with no active virus isolates after 2 days of symptom onset. Our viral culture data suggests that persistent PCR positivity may not correlate with infectivity, which has important implications for COVID-19 infection control precautions among older congregate care patients.

